# Optimization Method of Three-Dimensional Equilibrium Displacement in Thin Interbed Reservoirs

**DOI:** 10.1021/acsomega.3c05054

**Published:** 2023-11-17

**Authors:** Bin Jiang, Shiqing Cheng, Kang Ma, Qiao Guo

**Affiliations:** † China University of Petroleum (Beijing), Beijing 102249, China; ‡ China National Offshore Oil Corporation, Beijing 100010, China

## Abstract

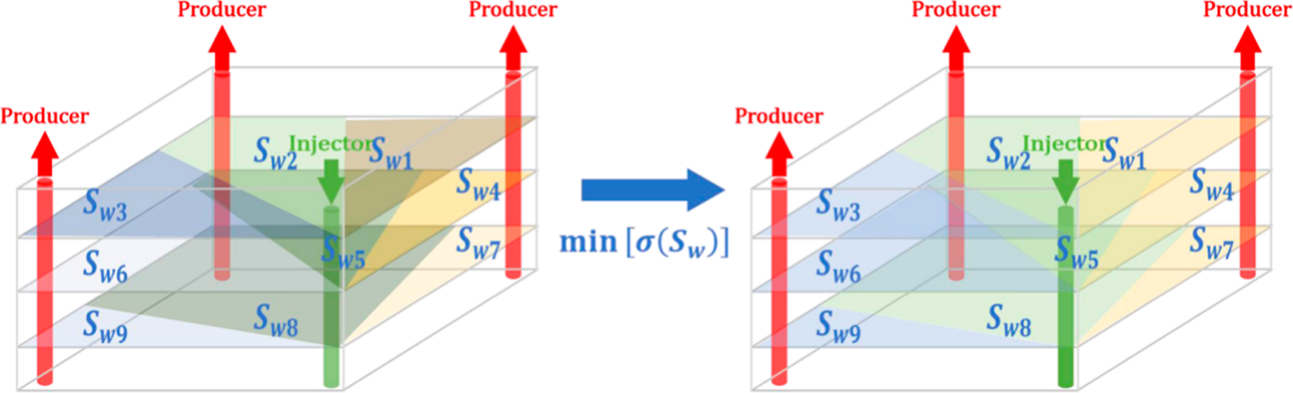

Most thin interbed reservoirs face a common problem that a nonequilibrium injection and production relationship in plane and vertical directions results in quick water breakthrough, rapid water-cut rise, and a poor water flooding efficiency in a single layer. A finer injection-production strategy should be developed to avoid serious water channeling and an ineffective water cycle. To narrow this gap, this work presents a three-dimensional intelligent equilibrium displacement model (3D-IEDM) to optimize water flooding in thin interbed reservoirs. A water-injection splitting model is first established to determine the water-injection rate of each layer based on displacement pressure and flow resistance. Then, water saturation is calculated for the injection-production well group based on the material balance principle. To achieve three-dimensional equilibrium flooding, the minimum water saturation variance is chosen as the optimization target and the improved particle swarm optimization algorithm is employed to reduce the optimization time caused by iterative calculations. Finally, the 3D-IEDM is programmed as software to provide a quantitative equilibrium flooding optimization scheme in an actual oilfield. The implementation in the pilot B36 well group test of the PL oilfield indicates that the optimization velocity of the 3D-IEDM can optimize the vertical water injection profile of thin interbed reservoirs and improve the sweep efficiency, and the length of time is approximately 14 times less than that of conventional simulator-based methods. Compared with the conventional injection-production scheme, the initial productivity of the pilot well group using the 3D-IEDM increases by 6.45%, and the utilization factor of water injection improves by 15%.

## 1 Introduction

Water flooding plays an important role in field development;^1−3^ however, the oil production of many oilfields has declined and entered a high water-cut period in recent years.^4−6^ To increase the production of high water-cut oilfields while reducing the production cost at a low oil price is an urgent problem.^7−10^ For example, the PL oilfield in China has many vertical layers and strong heterogeneity. The single-layer water emergence and water content increase rapidly due to the initial joint injection and recovery with large cross-sections. Through a large number of implementation results, the fine water injection adjustment strategy combined with vertical and horizontal planes can effectively suppress the ineffective water cycle in the high water-cut stage of multilayer reservoirs.

Previous studies have shown that the adjustment of the development scheme in the high water-cut period is mainly obtained through optimization to achieve equilibrium displacement,^11−13^ which means that the injected subsurface strata are displaced to the same extent in all directions with a fluid wave coefficient of 100%. Fully equilibrium displacement is an ideal condition and is influenced by geology, well network conditions, fluids, and other factors,^14−16^ thus cannot be fully achieved in actual reservoirs. In order to achieve the maximum recovery benefit of crude oil, a high equilibrium displacement should be achieved as much as possible. Scholars have proposed different optimization methods according to the characteristics of different reservoir development stages. Generally, these methods were mainly based on maximizing production, and a series of production plans were developed by numerical simulation, and the final optimal plan was derived by economic evaluation.^17−19^ However, the method has disadvantages, such as a large workload and a long work cycle.

To date, researchers have developed injection-production optimization models using different algorithms.^20−24^ Isebor and Durlofsky developed the particle swarm optimization–mesh adaptive direct search (PSO–MADS) hybrid optimization algorithm to determine optimal development and production plans considering the objective of maximizing oil recovery while minimizing water injection.^25^ Ahmadloo et al. develop a new diagnostic model with multivariate partial least squares (PLS), response surface methodology (RSM), and artificial neural network (ANN) to improve the quality of predictions.^26^ Zhang et al. proposed a method with mixed integer linear programming (MILP) to evaluate the pressure and flow sensitivity of water injection pipeline networks for large-scale oilfield water injection systems.^27,28^ The summary of previous optimization models is listed in Table 1. All the above research studies only focus on one direction of equilibrium, either plane or vertical, and cannot achieve three-dimensional optimization. Furthermore, few studies have been conducted on multilayer thin interbedded reservoirs, in which the problem of time-variant reservoir parameters such as permeability and porosity during water flooding is easier to cause. It might lead to a mismatched result with actual production.

**Table 1:** Summary of Optimization Models

type of model	objective of optimization
PSO–MADS	maximum oil recovery but minimize water injection
diagnostic model with PLS, RSM, and ANN	quality of performance prediction of water flooding for heavy oil reservoirs
MILP	pressure and flow sensitivity of water injection pipeline networks
multilevel framework with iterative sequential, SPSA, and commercial simulator	well placement and control optimization under fluid processing capacity constraints
augmented Lagrangian function and SGFD	production rate control of the reservoir

The purpose of this paper is to study a three-dimensional intelligent equilibrium displacement model (3D-IEDM) for water flooding reservoirs, which can take both the direction of plane and vertical equilibrium displacement into consideration. Besides, the different injection allocation schemes are widely discussed in an actual oilfield, and they have a bit of directory significance to the development of thin interbedded reservoirs.

## 2 Methodology

**Figure 1:**
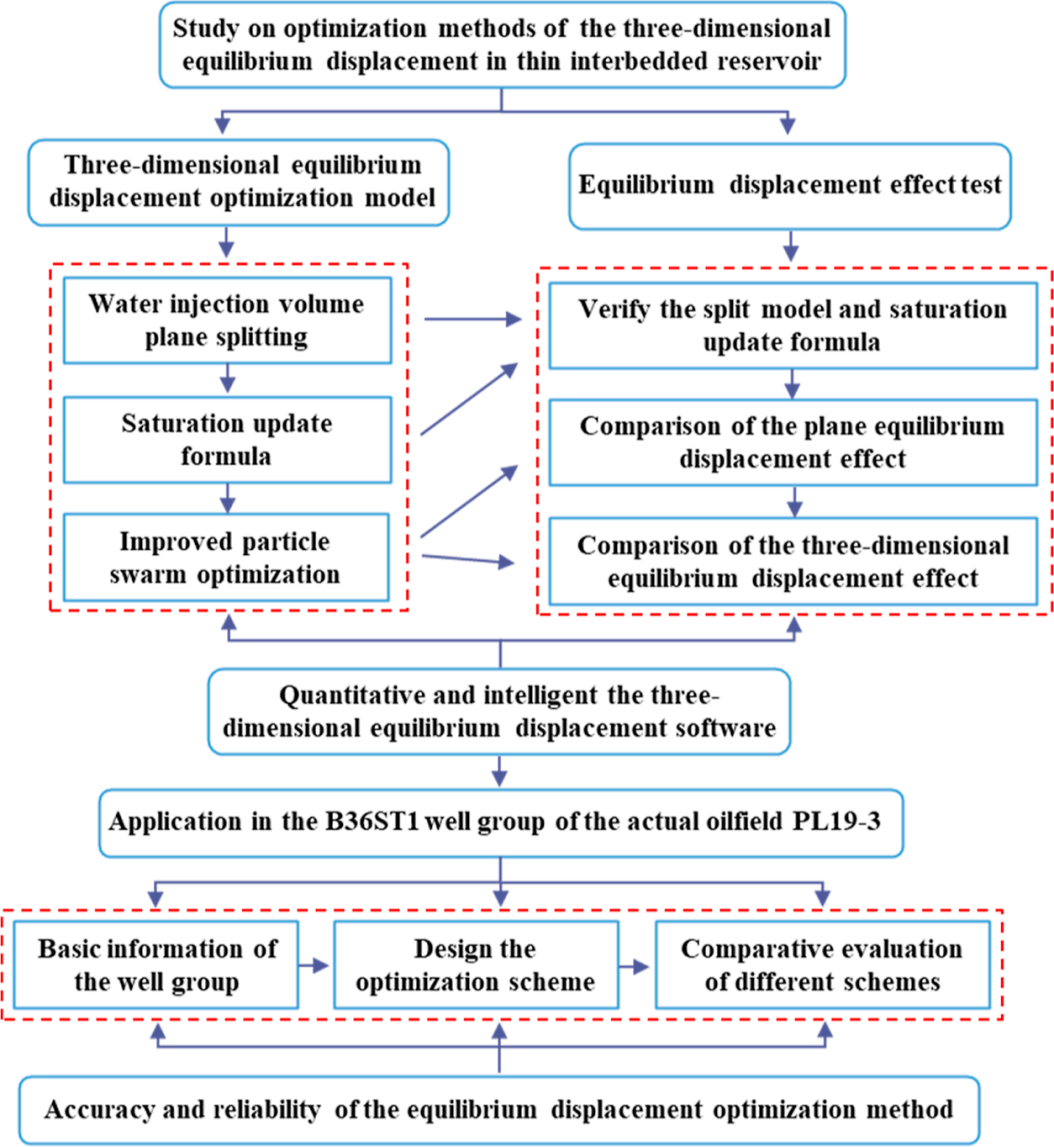
Research content of three-dimensional equilibrium displacement optimization.

A method for water injection splitting and injection-production well pair division was established first, and the quantitative relationship between the production dynamics of injection and production units and saturation changes was derived via reservoir engineering methods. Then, the mathematical algorithm was used to optimize the design scheme based on the saturation change in the injection-production unit, and a quantitative mathematical description method was developed with the three-dimensional equilibrium index as the optimization index. After that, the improved PSO algorithm was used for searching the optimal solution automatically. Finally, the 3D-IEDM will apply to one well group in the PL oilfield in Bohai Bay, China, for verification. The specific research content is shown in Figure 1.

### 2.1 Water Injection Splitting

**Figure 2:**
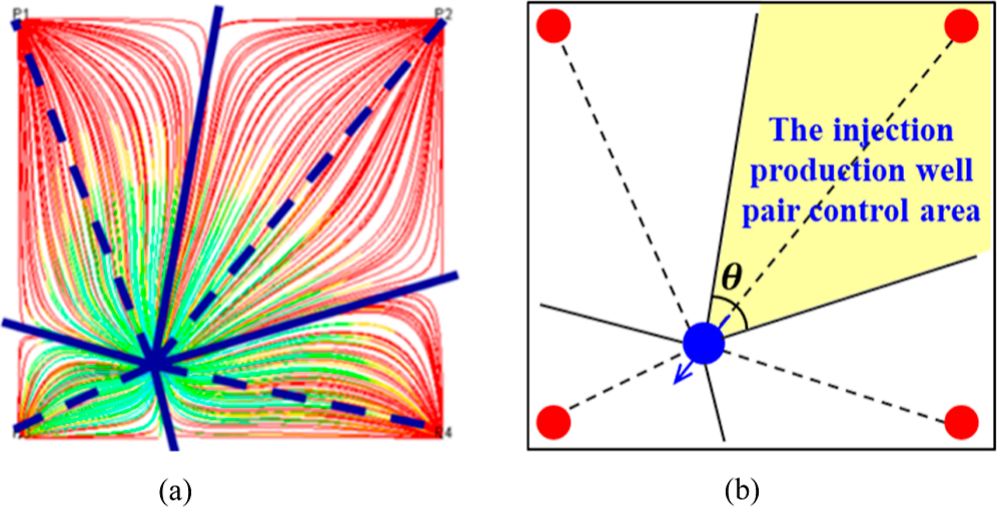
Two main methods of plane splitting.

The streamline simulation method is commonly used to calculate the splitting of water injection on the plane (Figure 2a). However, in practical applications, the history matching time is long, and the equilibrium displacement optimization scheme requires tens of thousands of iterations for calculation. Hence, the displacement pressure difference and flow resistance were employed to split the water injection volume, and the control area of different injection-production well pairs was divided according to the splitting ratio (Figure 2b).

Assuming that the compressibility of the reservoir and fluid is neglected, the displacement process involves only oil–water two-phase percolation and the temperature change during the displacement process is not considered. The fluid flow in the reservoir follows Darcy’s law. According to Darcy’s law, the displacement power between injection and production wells was controlled by the injection-production pressure difference. Under constant pressure, the production pressure difference could be determined directly. Under constant fluid production conditions, the flow pressure difference can be calculated from the fluid physical parameters between injection and production wells, as shown in eq 1, where the reservoir physical parameters are determined by the cross-well average value between wells. Since sand extraction was carried out under high fluid-production conditions, the production pressure differential in PL fields was generally set at 4 MPa,^29^ and the corresponding injection and extraction pressure differential was limited to about 6 MPa.
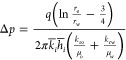
1

Actually, flow resistance is mainly affected by the crude oil viscosity, reservoir thickness, permeability, well spacing, relative permeability, and interwell connectivity, as well as the split angle.^30,31^ In order to improve the calculation accuracy, the well-pair control area was divided into two parts when the resistance in the high water-cut period was calculated. The oil saturation around the water well reaches the remaining oil saturation (0 ∼ *Lw*) and the area of oil–water two-phase flow (*L*–*L*_w_ ∼ *L*), which is calculated as shown in eq 2. On the basis of conventional reservoir calculation parameters, the influence of connectivity in thin interbedded reservoirs was increased by eq 2.
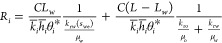
2

The splitting angle of different well pairs is shown in eq 3.
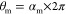
3

According to the power and resistance between injection and production wells, the splitting ratio of single-layer water injection wells in different directions and the corresponding water injection volumes were calculated (eqs 4 and 5).
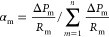
4
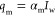
5

**Figure 3:**
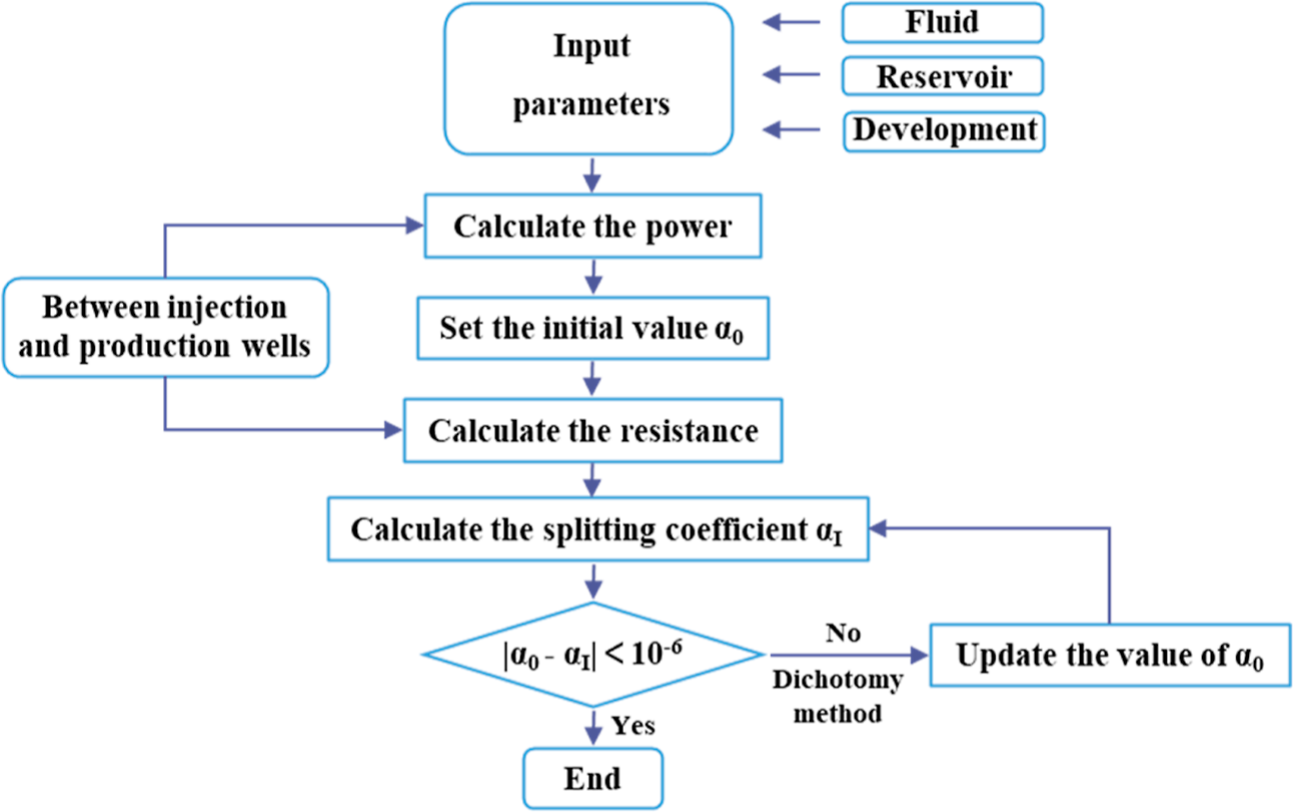
Specific calculation process of the iterative method.

Since the resistance calculation process involves an unknown splitting angle, an iterative method was adopted to solve the problem. The specific solution process is demonstrated in Figure 3.

### 2.2 Saturation Evaluation in Injection Production Units

After the split component of each water injection well was determined, the saturation change after water injection in the splitting unit was calculated through the material balance method. The oil–water flow in each injection-production unit obtained from the Darcy formula is shown in eqs 6 and 7.
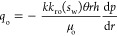
6
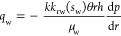
7

Equations 6 and 7 were added to get the total flow of oil–water flow, and the calculation equation is shown as eq 8.
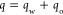
8

In order to simplify the calculation, eq 9 was used to combine the oil–water relative permeability. Therefore, the oil–water flow equation could be written as eqs 10 and 11.
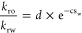
9
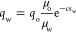
10
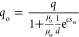
11

According to the material balance principle, oil production was increased from a single well at an underground water storage in the region, and the calculation equation is shown as eq 12.
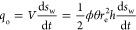
12

Equation 13 could be obtained by combining eqs 11 and 12, and the relationship between water saturation and time during production of the injection-production unit is shown in eq 13.
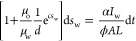
13

Equation 14 was obtained by integrating eq 13.
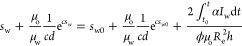
14

Equation 14 was rewritten into a Newton iteration form to obtain eq 15. Subsequently, eq 15 was transformed by a derivative to obtain eq 16. According to the Newton iteration method, the saturation update value was obtained as shown in eq 17.
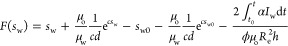
15
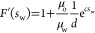
16
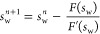
17

**Figure 4:**
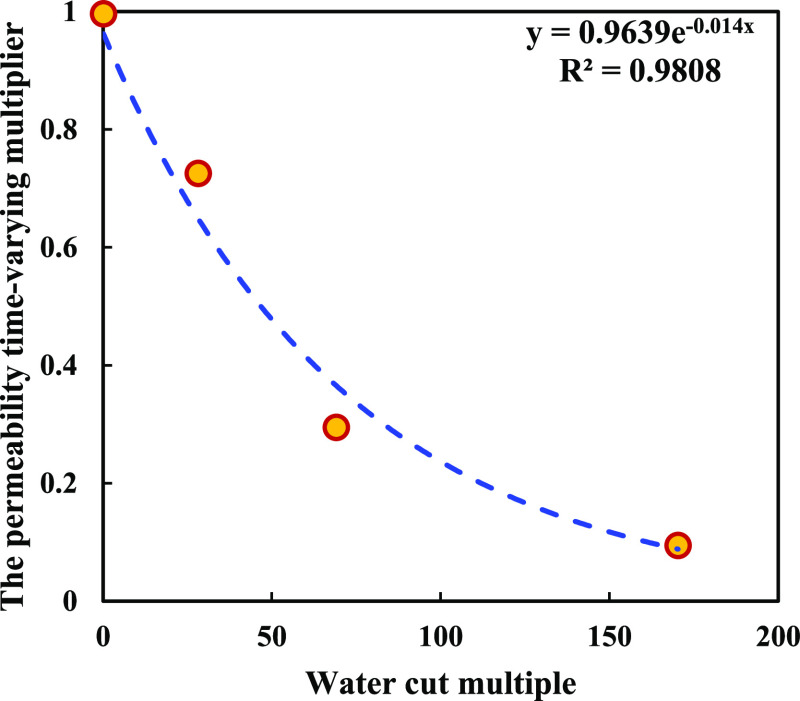
Variation curve between permeability change and water cut multiple.

During Newton’s iteration, the relationship between permeability variation and water content multiplier was derived from the statistics of the current well test interpretation results considering the development characteristics of sand emergence and plugging in the PL field, which have been validated by flow experiments^32^ (Figure 4).

In the actual calculation, the permeability time-varying multiplier was obtained by applying the regression equation in Figure 4, and the permeability value was updated by using eq 18, thus affecting the plane splitting of water injection.
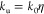
18

### 2.3 Quantitative Characterization of Equilibrium Displacement

In terms of optimization methods, the maximization of oil production was generally taken as the optimization goal, but the final optimization results of this method were achieved by strong injection and strong production, which promoted the production of high-yield liquid wells and aggravated the ineffective water cycle. Therefore, this paper proposes the minimum variance of saturation between injection-production well pairs as a three-dimensional equilibrium index, which is mathematically represented as shown in eq 19.
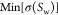
19

**Figure 5:**
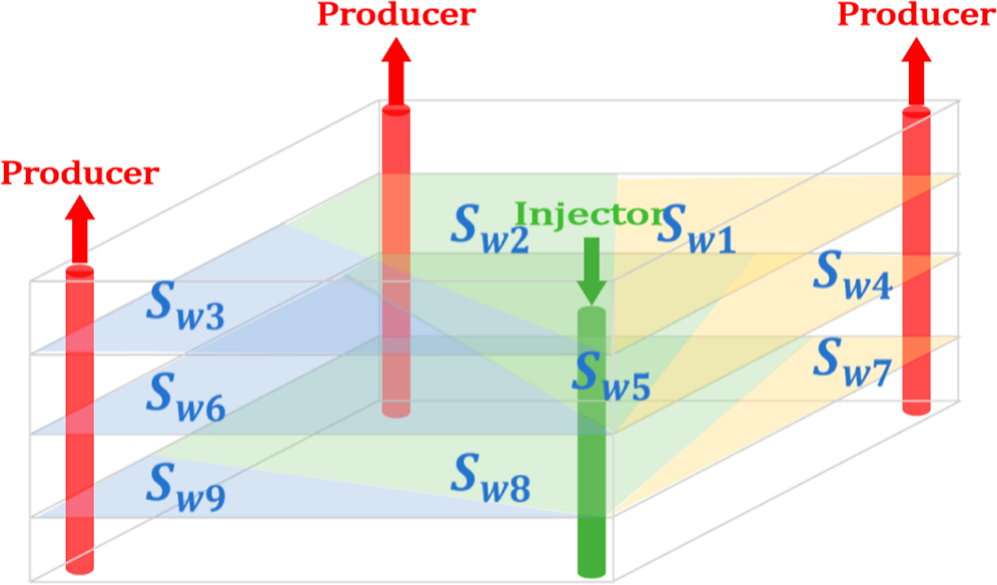
Injection and recovery well pair distribution model of a multilayer reservoir.

That is, attention is paid to the location of the production potential in the process of equilibrium displacement to improve the efficiency of water injection utilization. For single-layer reservoirs to achieve plane equilibrium displacement, adjusting the development dynamics of production wells can minimize the saturation variance. Compared with plane equalization, the goal of stereoscopic equalization optimization for multilayer reservoirs with separate injection and commingled production is to minimize the variance of saturation for all injection-production wells. The variables to be optimized are the single-layer splitting component of water injection wells and the development performance of the production wells. The distribution of injection-production well pairs is shown in Figure 5. The permeability of each layer is listed in Table 2, and there is no communication between layers.

**Table 2:** Values of Lateral and Vertical Permeability of Each Layer

layers	lateral permeability, mD	vertical permeability, mD
layer 1	300	30
layer 2	600	60
layer 3	900	90

In multilayer reservoirs, the water injection and liquid production of a single well are the sum of layered injection and production, respectively. The water injection and the liquid production of a single well are expressed in eqs 20 and 21, respectively. The saturation update equation for different flow units is shown in eq 22. In actual calculations, the number of injection-production well pairs is adjusted according to the plugging situation of layers.
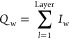
20
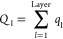
21
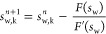
22

### 2.4 Improved PSO Algorithm

PSO is a new swarm intelligence computing technology proposed by Eberhart and Kennedy. It originated from the study of the movement behavior of birds and fish groups for solving complex optimization problems.^33,34^ PSO is initialized as a group of random particles, and then, the optimal solution is found by iteration. In each iteration, the particles update themselves by tracking two extremes. The first extreme value is the optimal solution found by the particle itself (i.e., the individual extreme value pBest), while another extreme value is the optimal solution found by the entire population, called the global extreme value gBest.

The position of particle *i* in the *N* dimension is denoted as *X*_*i*_ = (*x*_*i*1_, *x*_*i*2_, ... , *x*_*iN*_)^T^, the velocity is indicated as *V*_*i*_ = (*v*_*i*1_, *v*_*i*2_, ..., .,*v*_*iN*_)^T^, and the individual extremum is represented as *P*_*i*_ = (*p*_*i*1_, *p*_*i*2_, ..., *p*_*iN*_)^T^, which can be regarded as the particle’s own flight experience. The global extremum is expressed as *P*_*i*_ = (*p*_*g*1_, *p*_*g*2_, ..., *p*_*gN*_)^T^ and can be regarded as the group’s experience. The particles decide the next motion through their own experience and the experience of the group. For the *k* + 1th iteration, each particle changes according to eqs 23 and 24.
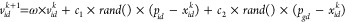
23
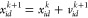
24

The inertia weight ω is a scale factor related to the previous speed. The larger ω and the smaller ω enhance the global detection ability and the local search ability of PSO, respectively. The calculation equation is shown in eq 25.
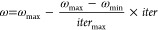
25

The standard PSO algorithm and various improved algorithms focus on how to make the particle swarm search more efficient in finding the optimal solution in the solution space. Nevertheless, in the later stage of the search, particles tend to be identical, which limits the search range of the particles. This paper adopts an improved particle swarm algorithm that uses the individual extremes of the preceding geese as the global extremes of the following geese; i.e., *p*_*id*_ and *p*_*gd*_ are replaced by *p*_*ad*_ and *p*_*a*(*i–*1)*d*_. The speed update and the position update are shown in eqs 26 and 27.
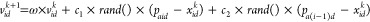
26
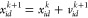
27

The individual extreme value of each goose except the head goose is transformed into the weighted average of its individual extreme value and its current fitness value *f*(*X*_*i*_), and the calculation formula is shown as equation eq 28.
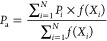
28

The improved PSO algorithm balances the contradiction between the search speed and accuracy. The particles use more information to decide their behavior, thereby reducing the probability of the algorithm falling into the local optimum. Moreover, the individual obtains greater incentives to enhance cooperation and competition among particles, thereby speeding up the convergence rate.

## 3 Results and Discussion

### 3.1 Numerical Model

**Figure 6:**
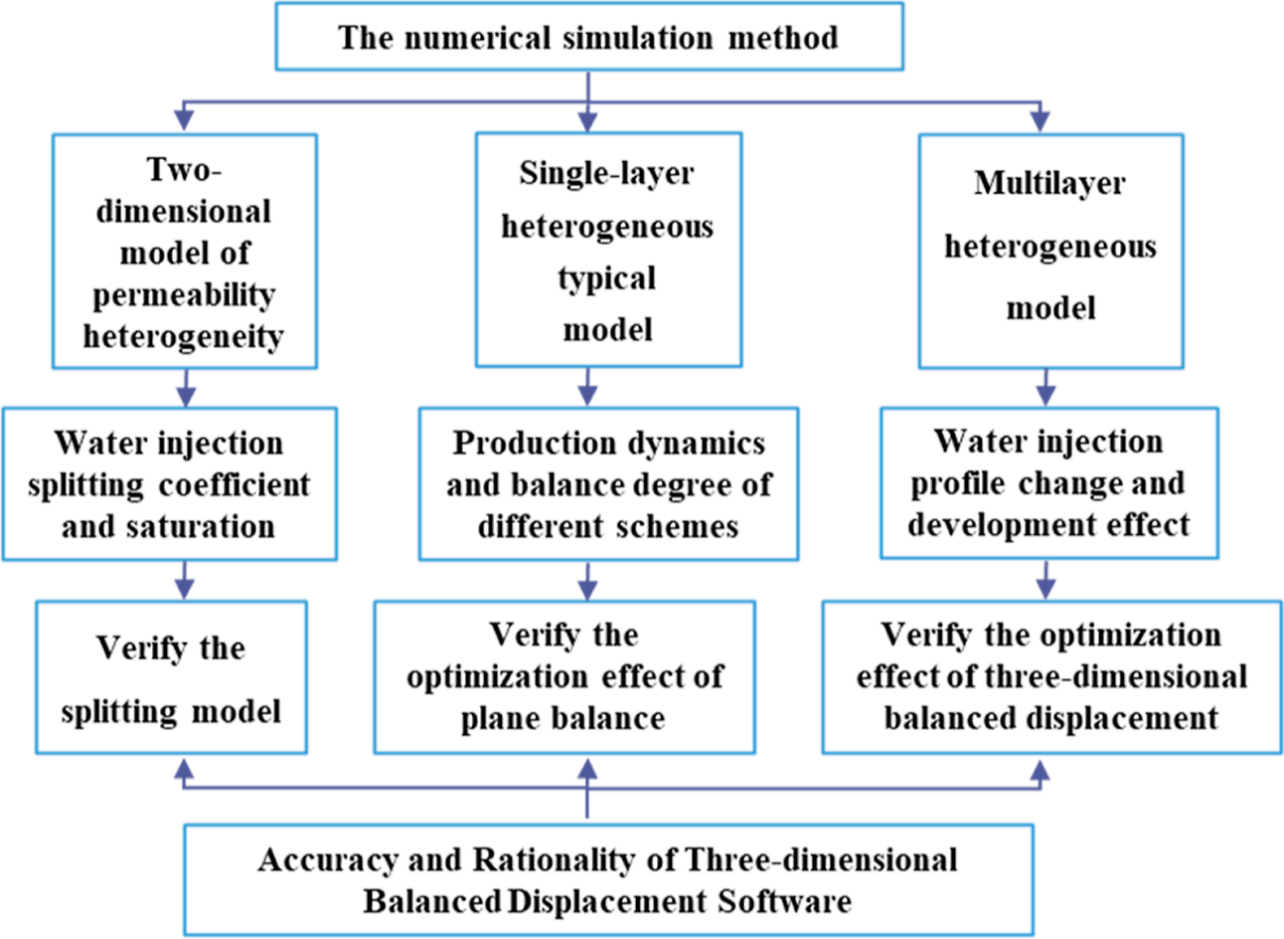
Research content of the numerical model.

In order to verify the rationality of the 3D-IEDM, the numerical simulation method was adopted to make an analysis and comparison. First, a two-dimensional model of permeability heterogeneity was established. The accuracy of the splitting model was verified by analyzing the splitting coefficient and saturation change of the water injection in different regions. A typical single-layer heterogeneous model was then established to verify the optimization results of the plane equilibrium index by comparing the production dynamics and equilibrium index of the original scheme and the equilibrium displacement scheme. Finally, a typical model of multilayer heterogeneity was established to achieve the splitting of vertical water injection and the optimization of plane oil well regulation. Furthermore, the effectiveness of stereo equilibrium displacement optimization was verified by comparing the change of water injection profile and development effect. The specific numerical model research content is depicted in Figure 6.

The numerical model was established by commercial numerical simulation software, and the injection-production system adopted the mode of one injection and four mining. The basic parameter information is shown in Table 3.

**Table 3:** Basic Parameter Information of a Single-Layer Model

mumber of grids	41 × 41 × 1	porosity (%)	25
grid size (m^3^)	10 × 10 × 2	bottom-hole flow pressure of water injection wells (MPa)	15
viscosity (mPa·s)	30	bottom-hole flow pressure of production well (MPa)	10
reservoir thickness (m)	10	number of perforated well sections	25

**Figure 7:**
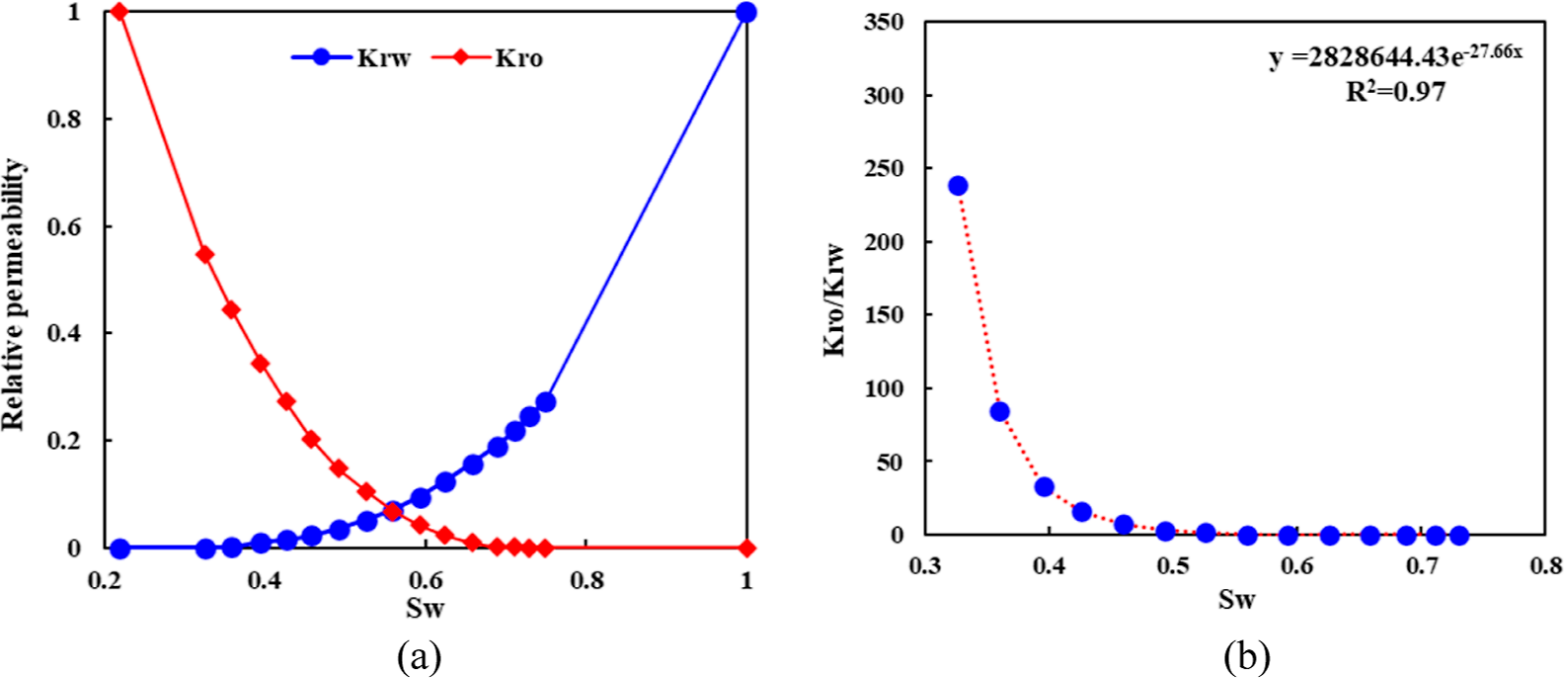
Oil–water relative permeability curve.

**Figure 8:**
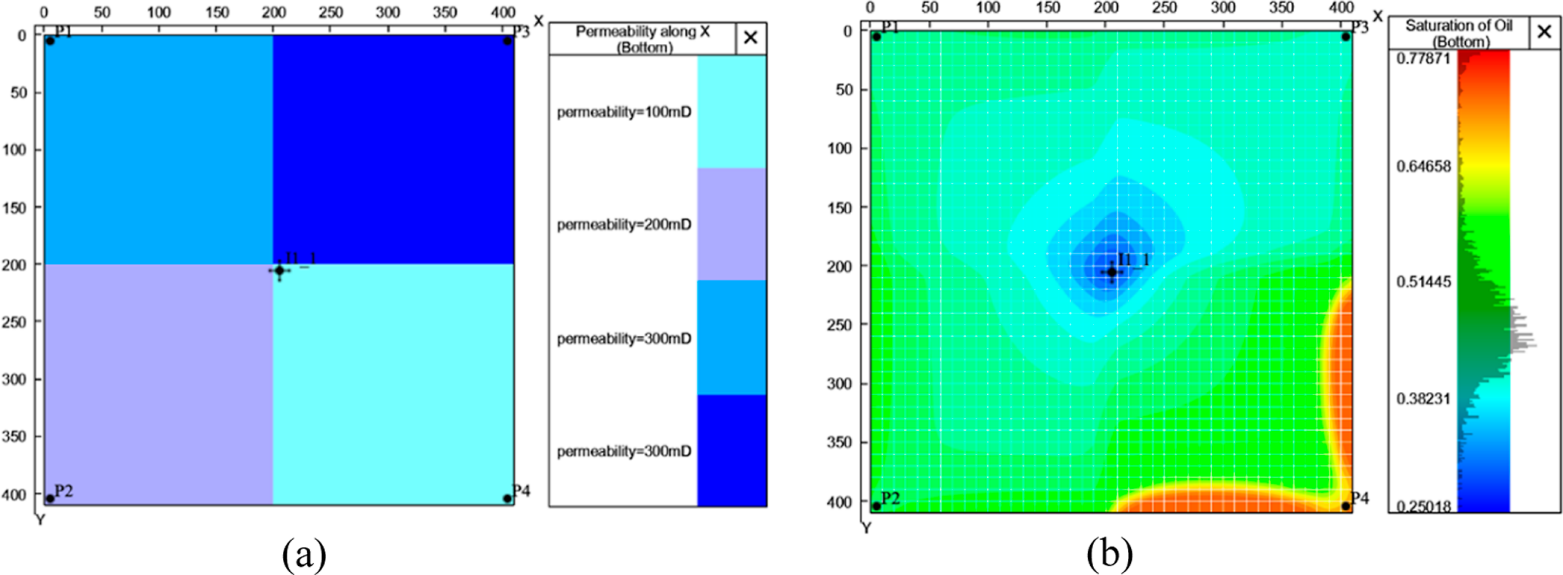
Basic information on the numerical model.

The oil–water relative permeability curve is described in Figure 7. The isotonic point on the relative permeability curve is 0.54, which indicates that the wettability of rock is hydrophilic, and the permeability field and residual oil distribution in the high water-cut stage are shown in Figure 8.

According to the permeability distribution field (Figure 8a), the permeabilities of oil-producing wells P1, P2, P3, and P4 are 100, 200, 300, and 300 mD, respectively. As the permeability was distributed in blocks, there was still remaining oil in the area with low permeability in this model, according to the distribution field of the remaining oil (Figure 8b).

### 3.2 Process of the 3D-IEDM

**Figure 9:**
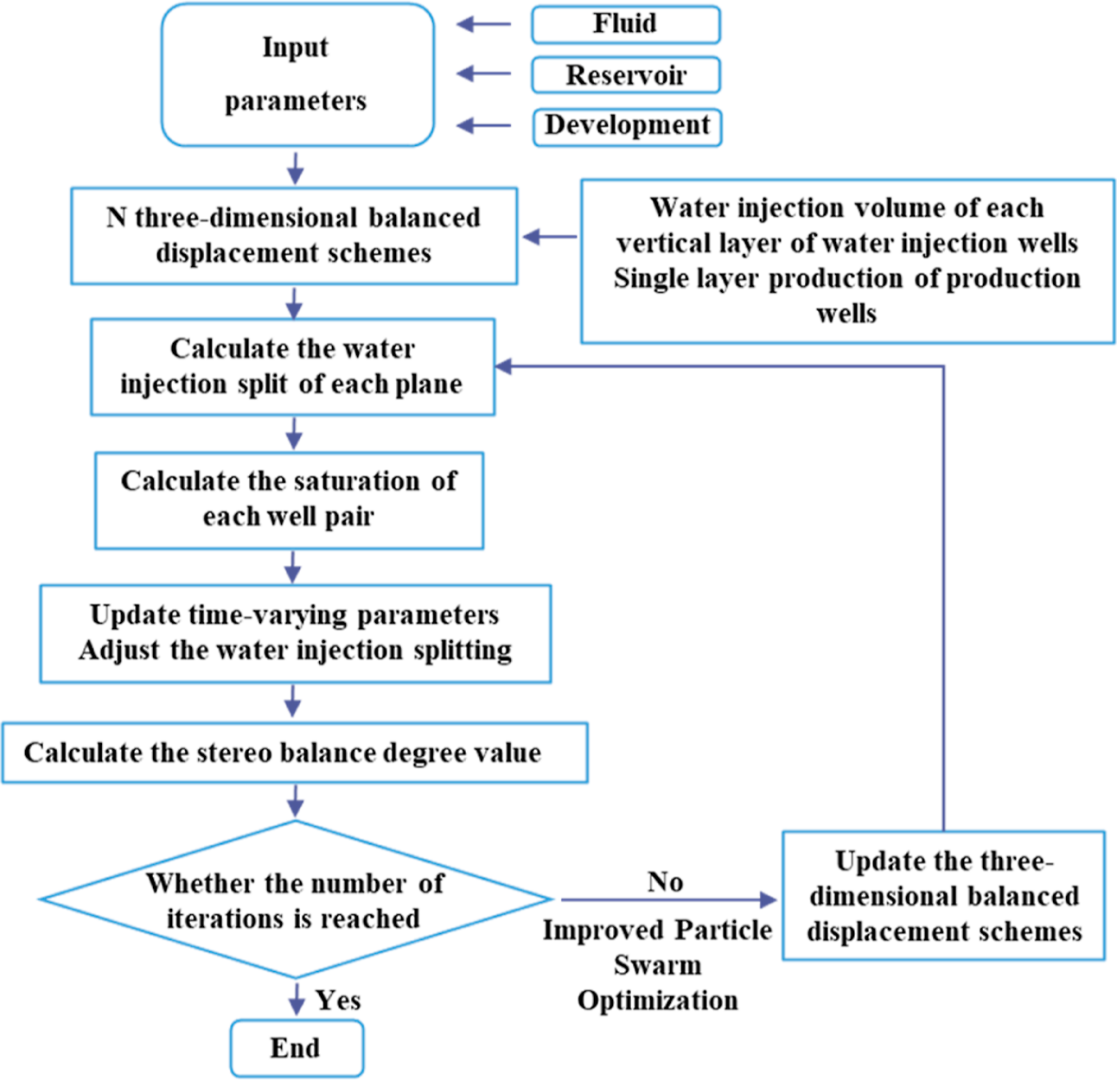
Detailed process of the 3D-IEDM.

Figure 9 shows a detailed process of establishing the 3D-IEDM. According to the parameters of fluid, geology, and production, numbers of three-dimensional equilibrium displacement schemes were randomly generated with the help of mathematical algorithms, including the vertical water injection volume of each layer of the water injection well and the single-layer production of the production well. The injection splits were calculated for each plane using an iterative calculation method, and then, the saturation of each well was calculated, the time-varying parameters were updated, and the injection splits were adjusted to obtain the three-dimensional equilibrium index. Finally, an improved PSO algorithm was utilized to update the vertical water injection volume of each layer and the single-layer production of production wells.

### 3.3 Equilibrium Evaluation of a Single Layer

Aiming to test the performance of plane equilibrium displacement, both the 3D-IEDM and numerical model were used to calculate the plane splitting ratio of injection wells and the saturation after 1 year of simulation between well pairs I-P1, I-P2, I-P3, and I-P4, respectively, within single layer models. The calculation results are shown in Table 4.

**Table 4:** Splitting Ratio and Saturation Comparison between the 3D-IEDM and Numerical Model

		well pairs			
model comparison		I-P1	I-P2	I-P3	I-P4
split ratio	3D-IEDM	33.13%	18.43%	43.35%	5.09%
	numerical model	33.84%	19.67%	41.54%	4.95%
saturation after 1 year	3D-IEDM	0.54	0.53	0.56	0.47
	numerical model	0.55	0.53	0.57	0.46

**Figure 10:**
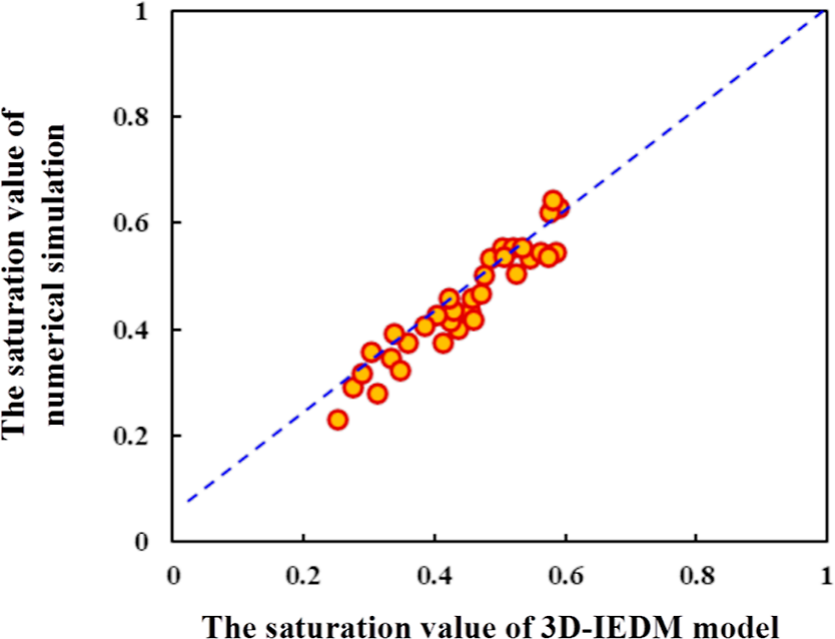
Comparison of the 3D-IEDM and the numerical simulation of 10 iterative samples.

The splitting proportion and saturation calculated by the 3D-IEDM were basically consistent with the numerical simulation results. To further verify the accuracy of the 3D-IEDM, 10 more samples were selected for comparison along with the iterative optimization process. The results are shown in Figure 10.

In Figure 10, the abscissa represents the saturation value of the well pair calculated by the 3D-IEDM, and the ordinate represents the numerical simulation result. It can be seen intuitively from Figure 10 that the calculation results of the 3D-IEDM and the numerical model basically approach different saturation ranges, with values mainly in the range of 0.2–0.6.

**Figure 11:**
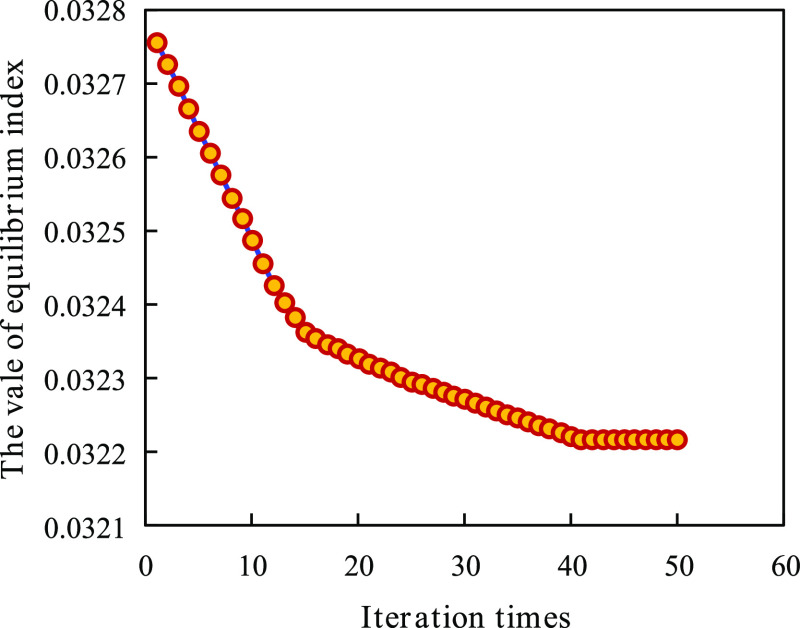
Value of the equilibrium index changes with the number of iterations.

The improved PSO was used to iterate 50 times with 50 schemes in each iteration. The changes in the equilibrium index in the optimization process are displayed in Figure 11. As can be observed from the figure, the change range of the value of the equilibrium index gradually becomes smaller and gradually approaches the optimal value with an increase in iteration.

Three cases were designed to demonstrate the rationality of optimization results using a numerical model (Table 5). In case 1–1, the bottom-hole flow pressure of 10 MPa was fixed in each producer as an original scheme. In case 1–2, the bottom-hole flow pressure of each well was recalculated by the 3D-IEDM. As shown in the table, P2 and P4 were reduced to 9 MPa (the maximum allowable range), and the values of P1 and P3 were also recalculated as 9.3 and 9.9 MPa, respectively, which means more water will be allocated between I-P2 and I-P4 well pairs to ensure high liquid production.

**Table 5:** Working System of Injection Production Wells in Different Schemes

		bottom-hole flow pressure of production well				
scheme name	water injection well pressure (MPa)	P1 (MPa)	P2 (MPa)	P3 (MPa)	P4 (MPa)	Scheme
case 1–1	15	10	10	10	10	original
case 1–2	15	9.3	9.0	9.9	9.0	equilibrium displacement
case 1–3	15	9	9	9	9	maximum liquid production

**Figure 12:**
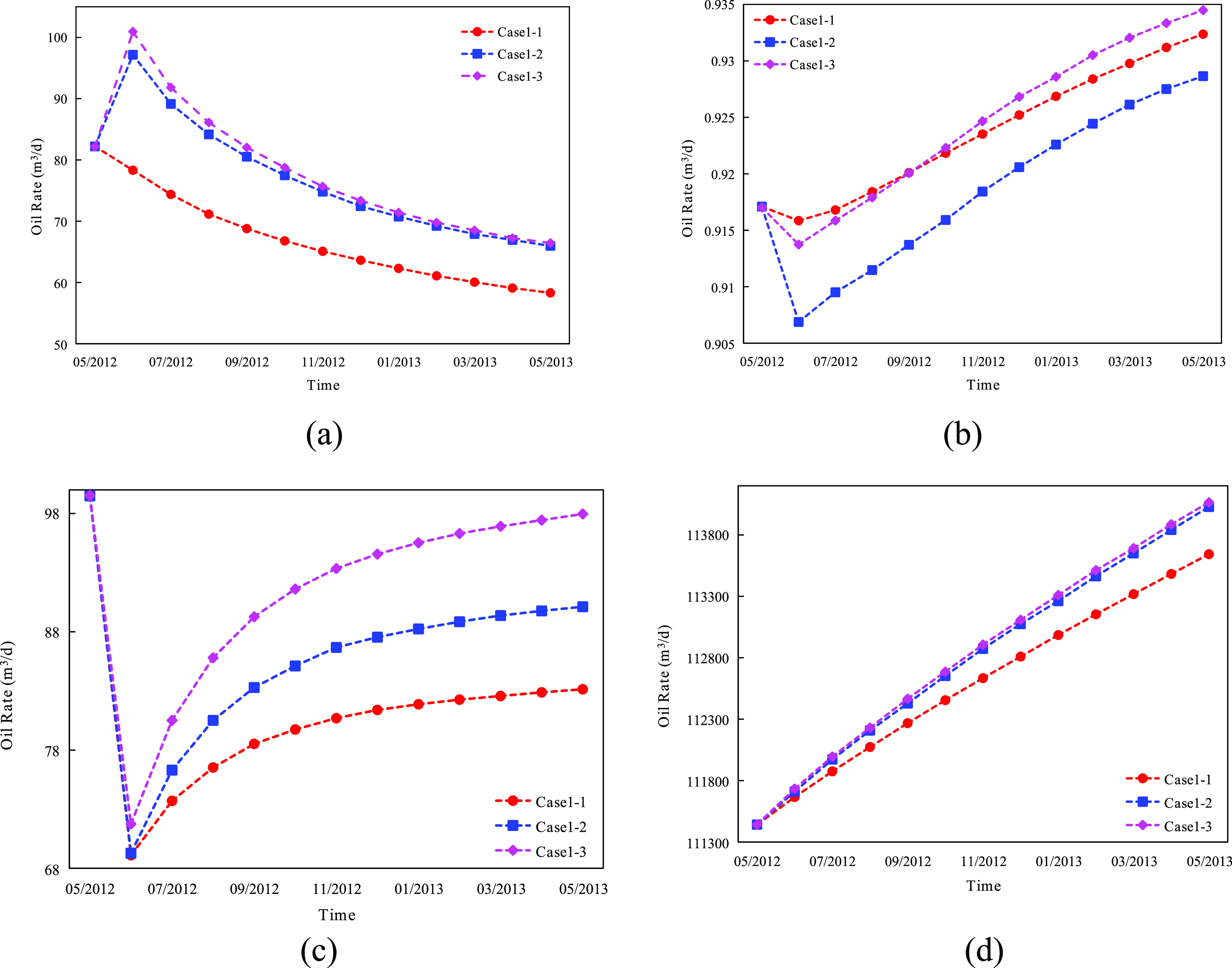
Comparison of production dynamics of different schemes within 1 year.

Case 1–3 is the maximum liquid production scheme, in which the bottom-hole pressure of all producers is set to the minimum value of 9 MPa. The case releases the differential pressure production and can intuitively compare the difference between the optimal equilibrium index and optimal oil production. The numerical model was adopted to obtain the production dynamic curve of each scheme within 1 year (Figure 12).

**Figure 13:**
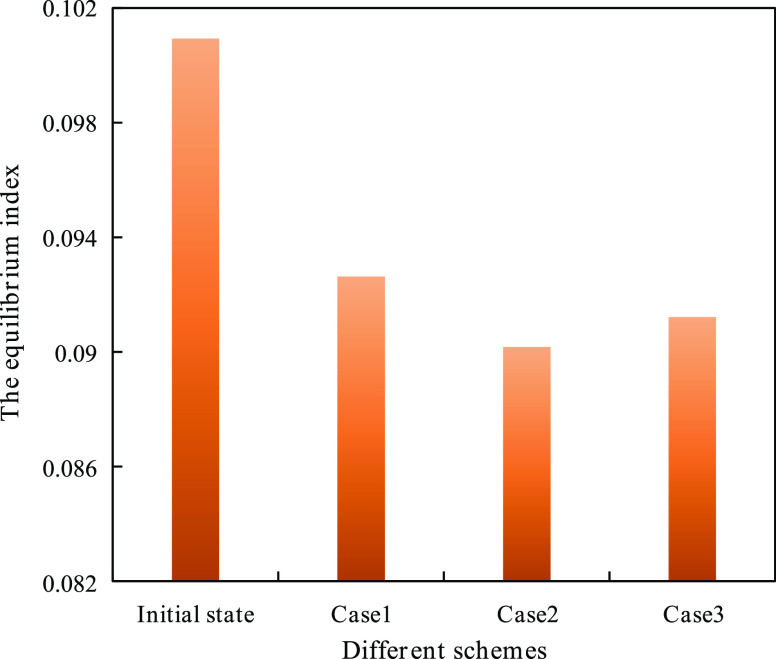
Comparison of the equilibria index of different schemes.

Compared with case 1–1, the productivity of case 1–2 increased by 24.92% in the first three months, the water cut decreased by 1.37%, the water injection efficiency increased by 2.88%, and the cumulative production increased by a total of 19.48% in 1 year. Compared with cases 1–3, cases 1–2 have similar production rates after six months of production but a slow increase in water content and a 0.18% increase in the water injection efficiency. One year later, the water cut decreased by 0.95%, and the final production rate of both scenarios was only 1.19%. The numerical simulation results show that cases 1–2 can maximize the use of injected water and improve the recovery efficiency. Grid saturation and variance were calculated for the equilibrium index of the three numerical models after 1 year. The comparison results are shown in Figure 13.

Notably, all of the equilibrium indexes of the three schemes were improved compared with the initial state, and the equilibrium displacement case reached the highest level.

### 3.4 Equilibrium Evaluation of Multilayers

**Figure 14:**
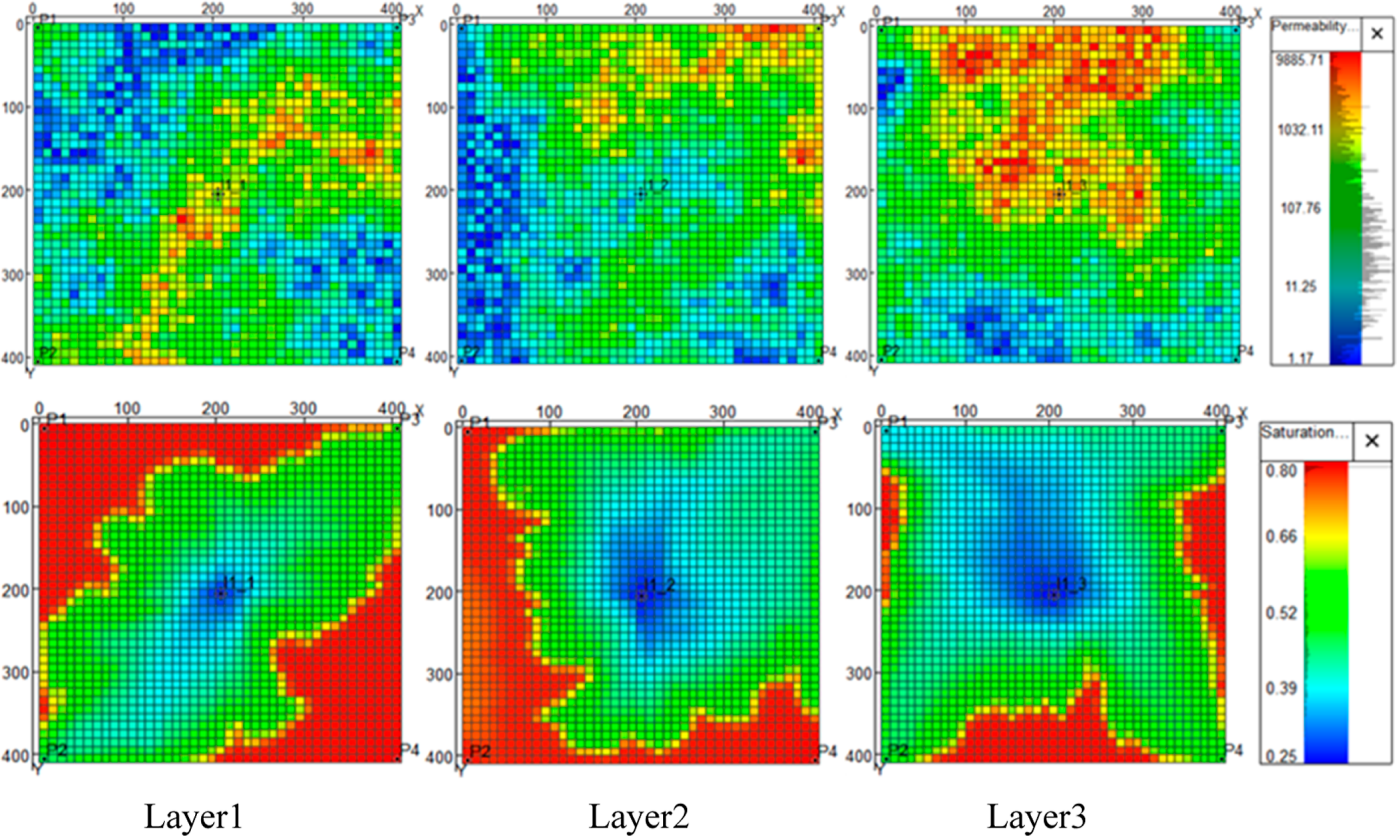
Basic information on the multilayer model.

A multilayer model with two more layers on the basis of a single-layer model was established to observe the performance of stereoscopic equilibrium displacement. It is worth noting that, besides production rate, injection allocation of water injectors is also one variable, which needs to be adjusted in the multilayer model. The basics of the multilayer model are shown in Figure 14.

**Figure 15:**
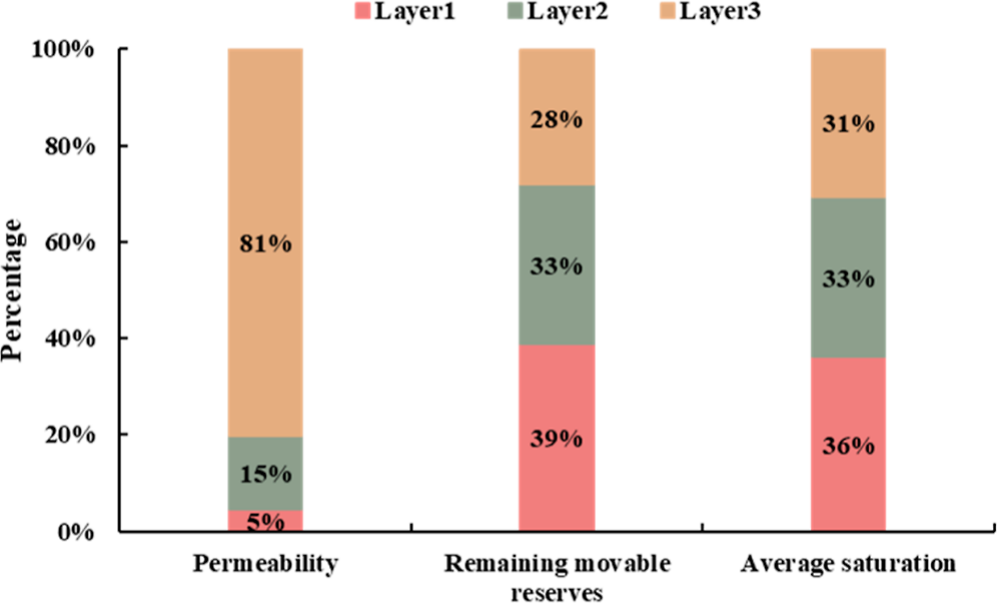
Comparison of the development status of different layers.

Figure 14 shows the distribution of permeability and saturation in three different layers. It should be noted that the third layer where P1 is located and the second layer where P3 is located have been flooded and have less development potential. The development effects of different layers vary greatly during combined injection development because of the serious heterogeneity of the reservoir. The fluids and rock properties of each layer are listed in Table 6. The comparison of the development status of different layers is shown in Figure 15.

**Table 6:** Values of Fluids and Rock Properties of Each Layer

layers	oil viscosity, cp	compressibility of rock, MPa^–1^
layer 1	30	0.000963
layer 2	30	0.000963
layer 3	30	0.000963

**Figure 16:**
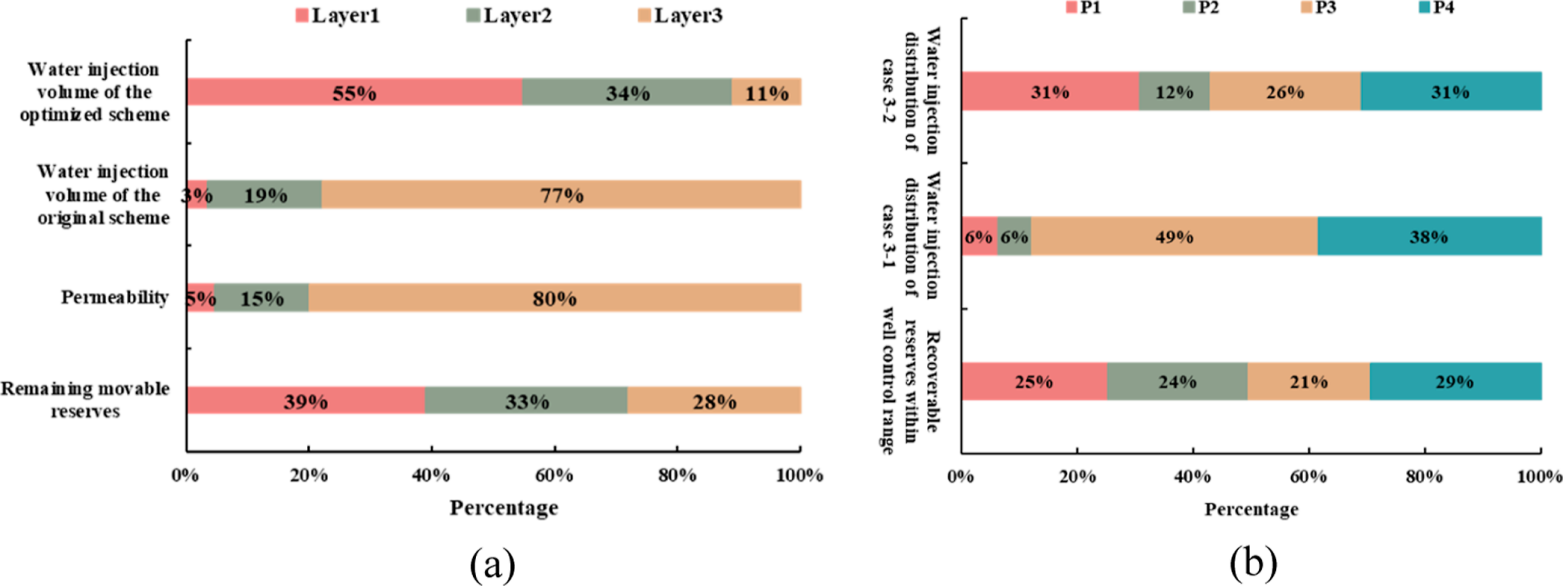
Water injection distribution of different layers and different water injection wells.

The proportional distribution of permeability, remaining movable reserves, and average saturation of different layers are shown in Figure 15. It can be seen from the figure that the three layers have strong interlayer heterogeneity. The current remaining reserves are mainly concentrated in the upper two layers. Two cases were conducted in the numerical model, in which case 3–1 stands for the equilibrium displacement scheme and case 3–2 represents the original scheme. The comparison of the results of injection wells between the two cases is shown in Figure 16.

**Figure 17:**
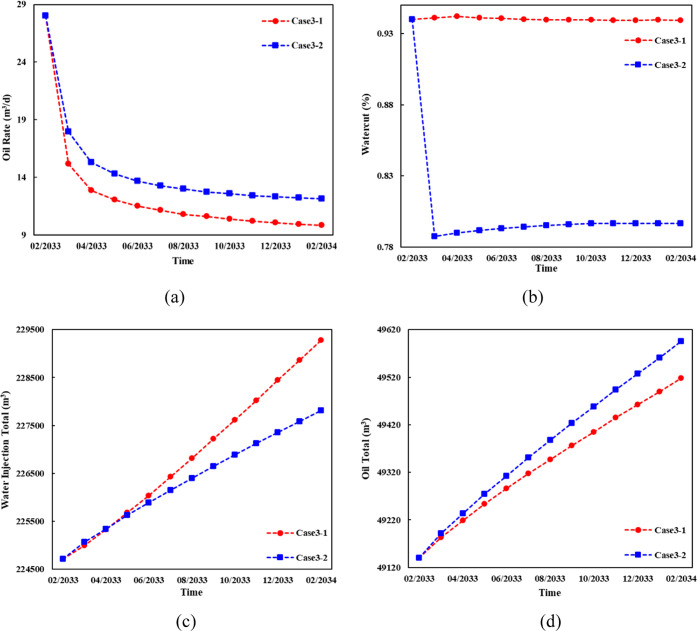
Comparison of production dynamics in the 1 year for different schemes.

The graph of the water injection distribution coefficients of production wells shows that case 3–2 distributes water injection mainly by increasing the water injection in layers 1 and 2 (Figure 16a). According to the distribution ratio of water injection of different production wells (Figure 16b), case 3–2 improves the split ratio of water injection wells of the P1 and P2 wells but reduces the splitting ratio of water injection wells of the P3 well. The splitting ratio of the P4 well is similar in two cases. The adjusted development effect is shown in Figure 17.

**Figure 18:**
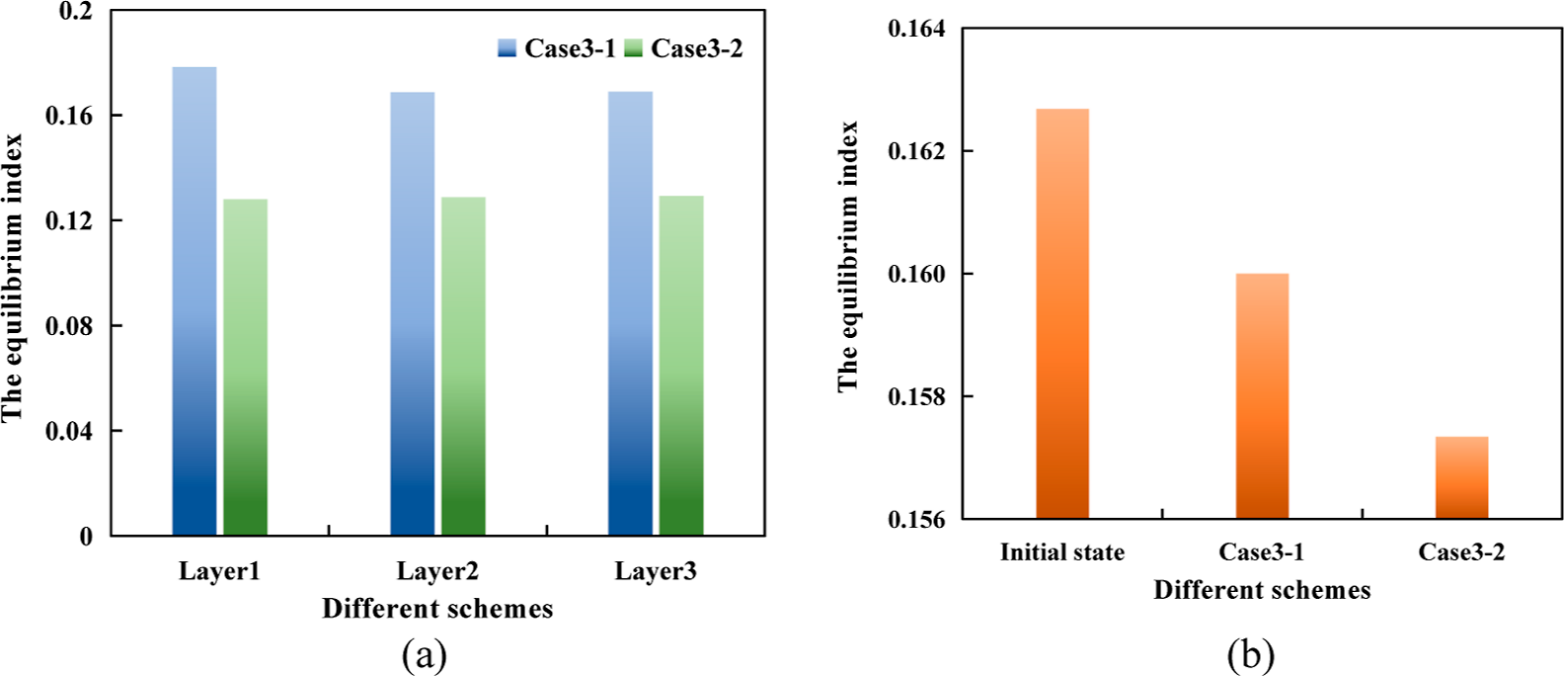
Comparison of the grid saturation equilibrium index for different schemes.

By adjusting the equilibrium scheme, the numerical model was used to predict the development effects of the two schemes within 1 year. From Figure 17, the capacity of case 3–2 increased by 9.34% in the first three months. Due to the closure of the high aquifer section, the water cut decreased by 15.31%, the water injection utilization rate increased by 6.52%, and the cumulative production within 1 year increased by 20.59%. It is noteworthy that case 3–2 produced more volume of oil with less water injected due to the high equilibrium index, which means more injected water participates in ineffective circulation in the reservoir in case 3–1. The equilibrium index of the two schemes was calculated, and the results are shown in Figure 18.

From the comparison diagram of the equilibrium index of different layers of the two schemes (Figure 18a), it can be concluded that the equilibrium displacement optimization scheme has the same equilibrium index in the three layers and is superior to the original scheme. From the overall comparison diagram of the model (Figure 18b), the equilibrium effect of the original scheme and the equilibrium displacement optimization scheme is better than that before the adjustment. The equilibrium displacement optimization scheme is better than the original scheme. Therefore, the equilibrium displacement scheme is superior to the original scheme with respect to the single-layer equilibrium effect or the overall equilibrium effect of the model.

### 3.5 Field Application

After verification with the numerical model, the 3D-IEDM was used to optimize the design of layer injection allocation of the B36 well group in the PL oilfield.

**Figure 19:**
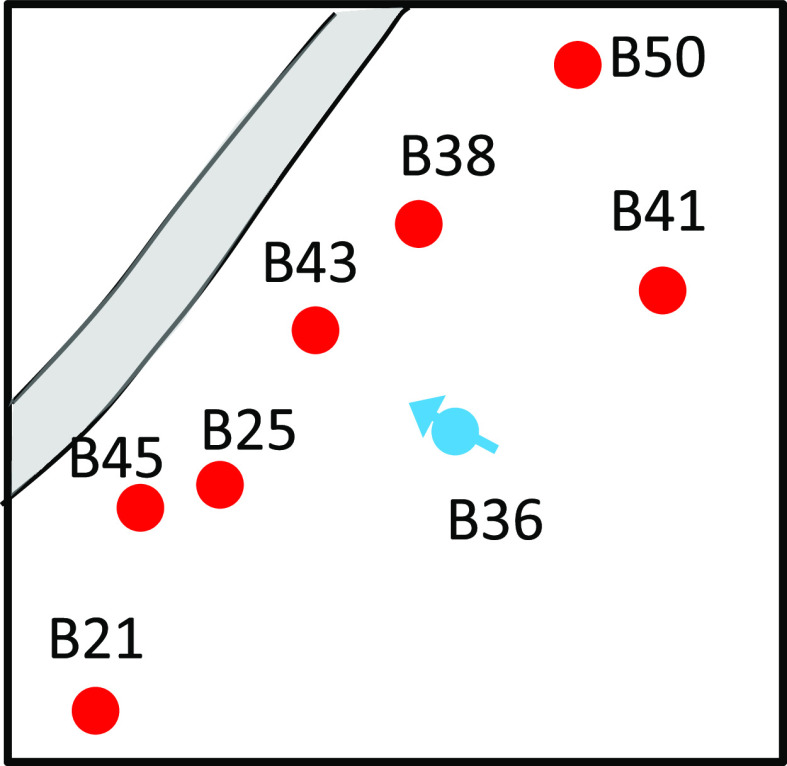
B36 well group’s one-layer well location distribution.

The B36 well was put into injection in December 2010, and water was injected to support 7 oil producers according to 7 sand control sections. The well location distribution is depicted in Figure 19.

**Figure 20:**
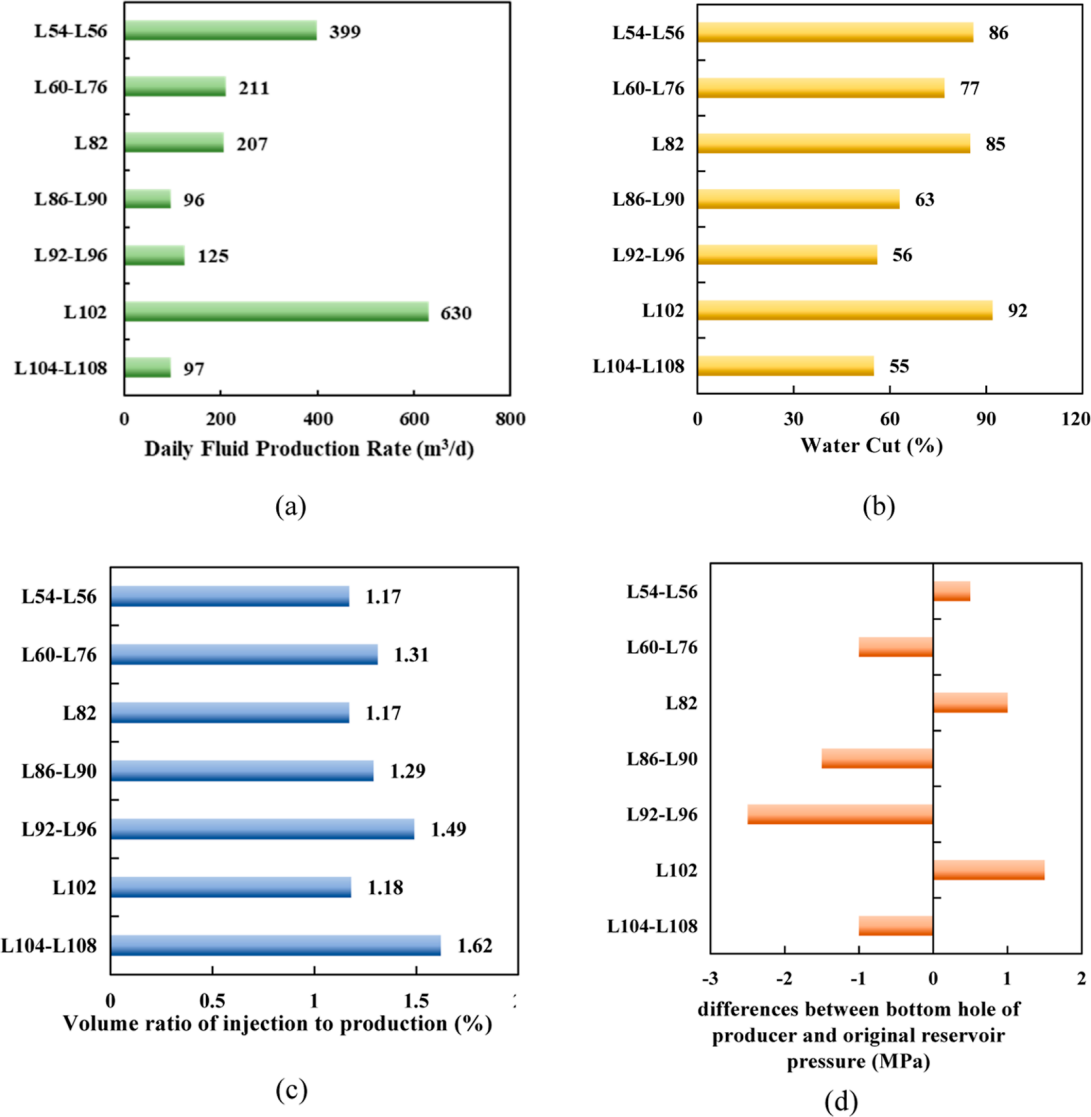
Production performance of sand control sections of the B36 well group.

Seven sand control sections are L54-L56, L60-L76, L82, L86-L90, L92-L96, L102, and L104-L108, and seven oil wells are B45, B25, B43, B39, B21, B50, and B41. It can be seen from Figure 19 that the distribution of oil and water wells in the B36 well group, in which the water cut of 7 wells is 62, 85, 86, 86, 94, 86, and 75%. The production performance of the B36 well group is described in Figure 20.

It can be concluded from the liquid production situation that the liquid production capacities of the oil wells in the L60-L76, L86-L90, and L92-L96 layers have not been fully utilized at the high liquid production and water content of the L102 layer with obvious water channeling. The test results show that even though all the layers of volume ratio of injection and production are greater than 1, there is underpressure in the L60-L76, L86-L90, L92-L96, and L104-L108 layers with poor injection and recovery connectivity. However, there is overpressure in the L102 layer because of good connectivity between producers and injectors.

**Figure 21:**
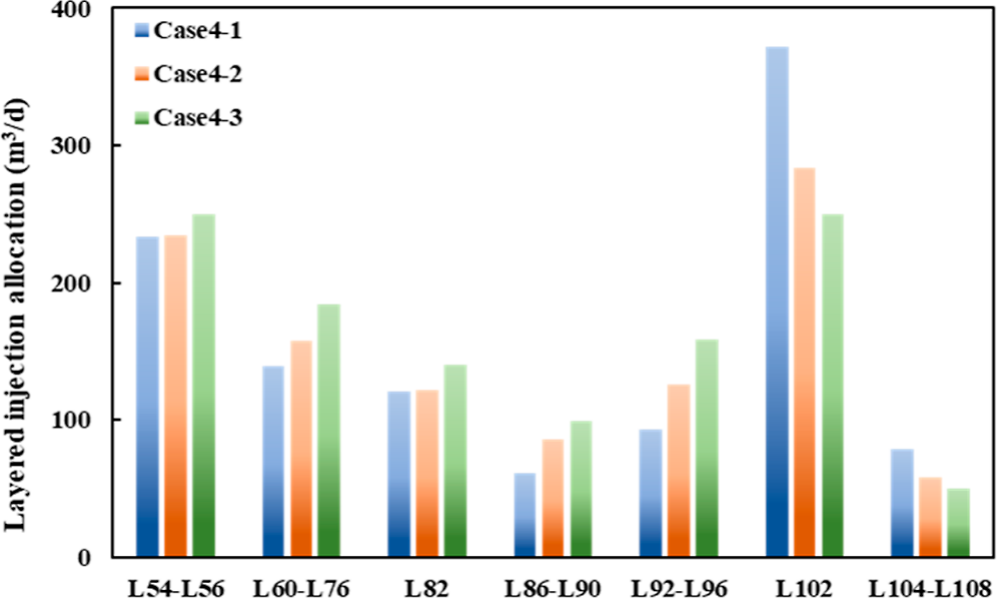
Layered injection allocation of different schemes in the B36 well group.

Three cases are taken into consideration for comparison in numerical simulation according to the reservoir physical properties and remaining oil distribution of each layer of the B36 well group, in which case 4–1 is the original injection scheme and case 4–2 and case 4–3 are optimization schemes with the conventional method and the 3D-IEDM. The injection allocation rates of different layers of 3 cases are shown in Figure 21.

**Figure 22:**
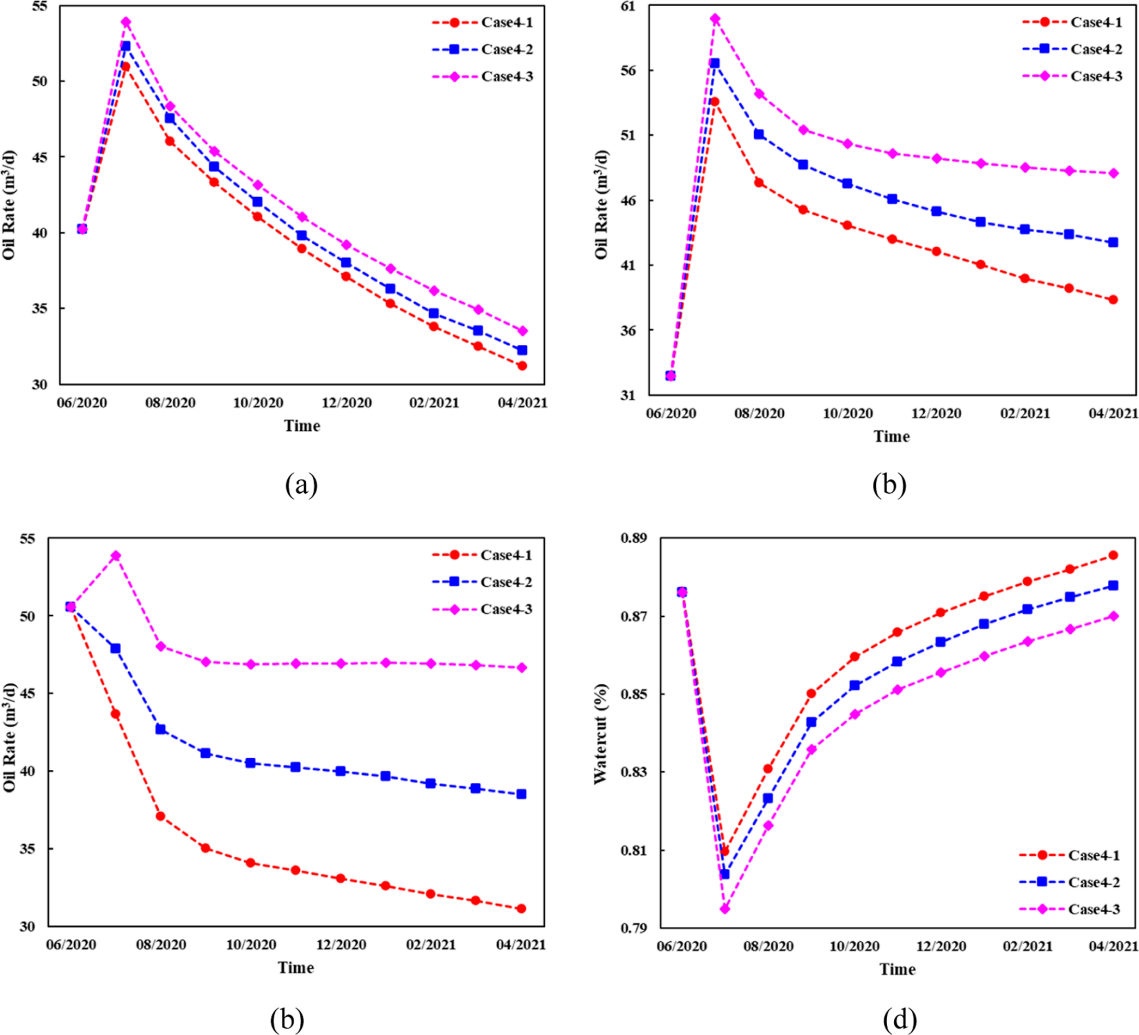
Production performance of adjustment schemes for the B36 well group.

As described in Figure 21, three schemes have different injection allocations for each layer, with less injection allocation in L86-L90 and more in L102. Case 4–3 limits the water injection of the L102 layer and enhances the water injection of the L86-L90 and L92-L96 layers. The development effect of different schemes is displayed after 1 year simulations for 3 cases in Figure 22.

**Figure 23:**
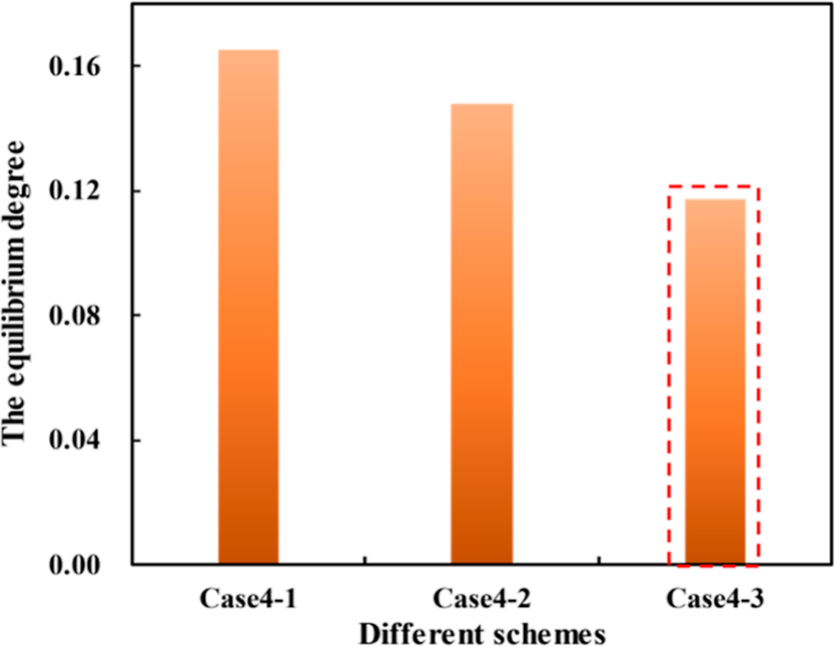
Equilibrium index of adjustment schemes of the B36 well group.

Both case 4–3 and case 4–2 increase the oil production of adjacent wells, and the daily oil increase of case 4–3 is 6.45% higher than that of case 4–2 (Figure 22) because more water is injected into the L86-L90 and L92-L96 layers with abundant remaining oil. Compared to case 4–1, the water cut of both case 4–2 and case 4–3 was greatly reduced without less water channeling in L102. The grid saturation variance of different schemes was calculated to obtain different equilibrium indexes. The calculation results are listed in Figure 23.

As can be seen in Figure 23, case 4–3 has an overall equilibrium index of the 3D-IEDM better than that of the conventional method and the original scheme, and the equilibrium index of the 3D-IEDM was increased by 15%.

**Figure 24:**
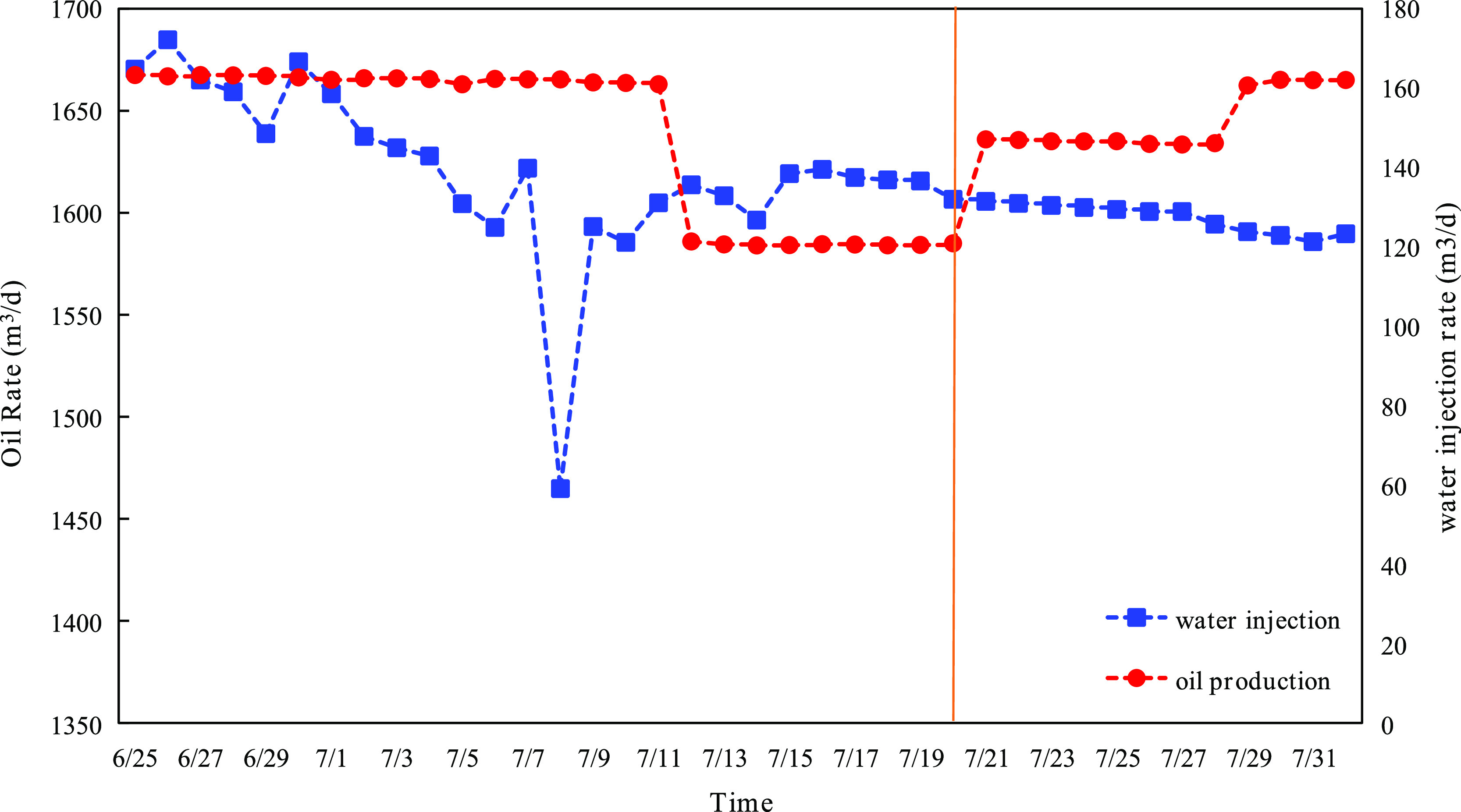
Actual oil rate and injection rate of the B36 well group after case 4–3 applied.

**Figure 25:**
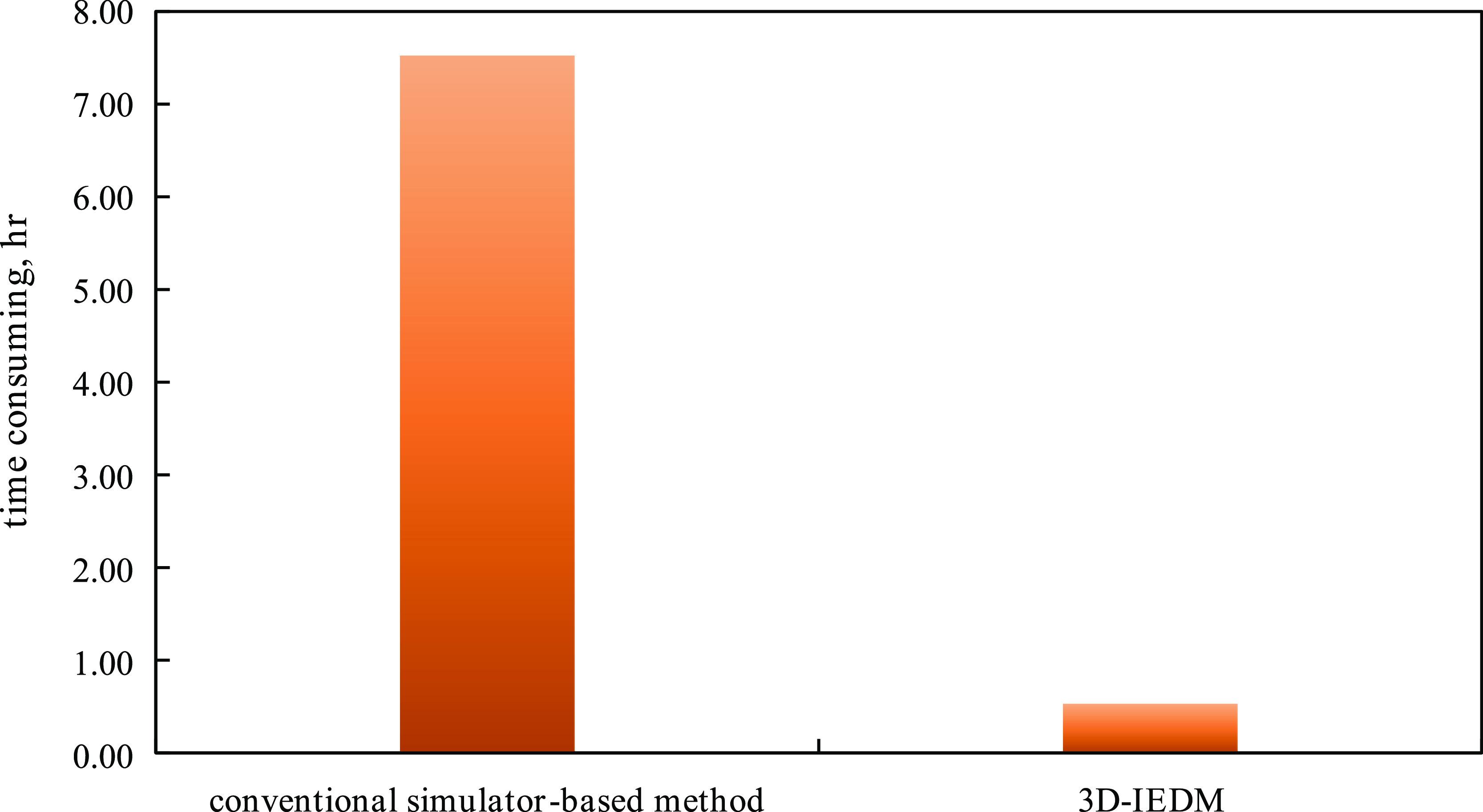
Comparison of the time consumption between the conventional simulator-based method and 3D-IEDM.

Finally, case 4–3 was selected to apply to the actual oilfield on the 20th of July; the oil rate and injection rate of the B36 well group are shown in Figure 24. As seen in the figure, the water injection rate was reduced slightly after optimization, and the oil rate of the well group was increased by about 25 m^3^/d (around 22.5%) than that before, which showed the validity of this method. The time consumption of the optimization is 0.53 h, which is 14 times less than that of the conventional simulator-based method; the comparison between the two methods is shown in Figure 25.

## 4 Conclusions

1 A new equilibrium displacement optimization model (3D-IEDM) is proposed for thin interbed water flooding reservoirs. The saturation variance of multilayers between producers and injectors was used as a quantitative objective to be optimized with an improved PSO algorithm.
2 The 3D-IEDM can provide an equilibrium displacement optimization scheme in a short period of time. Both the numerical model and actual field application proved that they can improve the degree of equilibrium of water flooding and the recovery of remaining oil more effectively than the conventional simulator-based method with a maximum liquid production constraint.
3 This model is useful to simulate the injection volume allocation of water injectors for thin interbed reservoirs under the condition of vertical wells. Further work needs to be done on the consideration of horizontal wells in thicker layers.

## Nomenclature Abbreviations

PSO: particle swarm optimization
3D-IEDM: three-dimensional intelligent equilibrium displacement model
PSO–MADS: particle swarm optimization–mesh adaptive direct search
PLS: partial least squares
ANN: artificial neural network
MILP: mixed integer linear programming
SPSA: simultaneous perturbation stochastic approximation
SGFD: stochastic gradient and the finite difference

## Symbols

Δ*p*: production pressure differential, MPa
*q*: flow, m^3^/d
*r*_e_: investigation radius, m
*r*_w_: wellbore radius, m
average permeability, 10^–3^ μm^2^
average thickness of the reservoir, m
*k*_ro_: oil-phase relative permeability, dimensionless dimension
*K*_rw_: water-phase relative permeability, dimensionless dimension
μ_o_: oil viscosity, mPa·s
μ_w_: water viscosity, mPa·s
*R*_*i*_: flow resistance, MPa/(m^3^/d)
*C*: connectivity coefficient, the value is between 0 and 1, dimensionless dimension
*L*_w_: distance to the residual oil area, m
*L*: well distance, m
θi*: the splitting angle, °
*s*_we_: water saturation under residual oil conditions, %
θ_m_: the splitting angle of number m well pair, °
α_m_: plane splitting ratio of water injection volume for number m well pair, fraction
*n*: number of well pairs in a single layer, dimensionless dimension
*I*_w_: single-layer water injection, m^3^/d
*m*: the number of well pairs, the value is between 1 and *n*, dimensionless dimension
Δ*p*_m_: production pressure differential of number *m* well pair, MPa
*R*_m_: flow resistance of number m well pair, MPa/(m^3^/d)
*q*_m_: calculated injection volume after splitting, m^3^/d
*q*_o_: flow of oil phase, m^3^/d
*q*_w_: flow of water phase, m^3^/d
*r*: equivalent control range, m
*c,d*: fitting coefficient of relative permeability, dimensionless dimension
*V*: the pore volume within the control range of the well pair, m^3^
*F*(*s*_w_): newton iterative function
*F’*(*s*_w_): derivative value of Newton iterative function
*s*_w_^*n*^: water saturation of the *n*th iteration step, %
*k*_0_: initial permeability, 10^–3^ μm^2^
η: permeability time-varying multiplier, dimensionless dimension
*k*_u_: updated permeability by time-varying multiplier, 10^–3^ μm^2^
σ(*S*_w_): variance of saturation between injection-production well pair, dimensionless dimension
*Q*_w_: water injection volume of water injectors, m^3^/d
*Q*_l_: production of producers, m^3^/d
layer: number of layers of the multilayer reservoir, dimensionless dimension
*s*_w,k_^*n*^: water saturation of the *k*th injection-production well pair at the *n*th iteration step,%
*i*: *i* = 1, 2,·..., *M*, *M* is the total number of particles in the population, dimensionless dimension
*d*: *d* = 1, 2,·..., *N*, *N* is the number of independent variables, dimensionless dimension
*c*_1_, *c*_2_: they are the acceleration factors, which adjust the maximum step size of flying in the pBest and gBest directions, respectively, fraction
*rand*(): random numbers between 0 and 1, fraction
ω: inertia weight, dimensionless dimension
ω_max_: the maximum value of ω, dimensionless dimension
ω_min_: the minimum value of ω, dimensionless dimension
*iter*: current number of iterations, dimensionless dimension
*iter*_max_: maximum number of iterations, dimensionless dimension
*X*_*i*_: the position of particle *i*, dimensionless dimension
*V*_*i*_: the velocity of particle *i*, dimensionless dimension
*P*_*i*_: the individual extremum of particle *i*, dimensionless dimension
Pg: the global extremum of all particles, dimensionless dimension
*f*(*X*_*i*_): the variance function of *X*_*i*_, dimensionless dimension
*P*_*ai*_: the improved individual extremum of particle *i*, dimensionless dimension
